# Dimethyl fumarate reduces the risk of mycotoxins via improving intestinal barrier and microbiota

**DOI:** 10.18632/oncotarget.17886

**Published:** 2017-05-16

**Authors:** Ning Ma, Yi Wu, Fei Xie, Kexin Du, Yuan Wang, Linxin Shi, Linbao Ji, Tianyi Liu, Xi Ma

**Affiliations:** ^1^ State Key Laboratory of Animal Nutrition, China Agricultural University, Beijing 100193, China; ^2^ Departments of Internal Medicine and Biochemistry, University of Texas Southwestern Medical Center, Dallas, TX 75390-9113, USA

**Keywords:** dimethyl fumarate, ultraviolet radiation, microbial composition and distribution, intestinal barrier function, mycotoxin

## Abstract

The effects of dimethyl fumarate (DMF) on mycotoxins and animal growth performance are well documented. However, its mechanism of anti-mildew effects is still unknown. The current study investigated how DMF detoxified the mycotoxin and improved the growth performance using BALB/c mice model, especially its effects on intestinal barrier function and gut micro-ecology. Our study also compared with the ultraviolet radiation (UR) treatment, a traditional anti-mildew control (TC). The results indicated that the DMF treatment had a lower contents of mycotoxin, better growth performance and improved mucosal morphology (*P* < 0.05), accompanied with the decreased intestinal permeability and the tighter gut barrier. Moreover, the efficiency of DMF was better than TC (*P* < 0.05). 16S rRNA gene sequence analysis revealed that the richness and diversity of bacteria was increased in DMF treatment. The most abundant OTUs belonged to Firmicutes and Bacteroidetes, and their changes in DMF were more moderate than the TC group, suggesting a more stable micro-ecology and the positive impact of DMF on the biodiversity of intestine. Specifically, the increased abundance of bacteria producing short-chain fatty acids (SCFAs), such as *Gemella, Roseburia*, *Bacillus* and *Bacteroides* in DMF group and prebiotics such as *Lactobacillus* in TC group, suggested a more healthier microbial composition and distribution. These findings supported that DMF had significant effects on animal's growth performance and intestinal barrier function by modulating the pathway of nutrient absorption and increasing the diversity and balance of gut microbes, which also illuminate that DMF is more efficient than traditional anti-mildew method.

## BACKGROUND

Attentions have focused on mycotoxins for their infection on monocotyledon-based crops such as maize, wheat and barley [1] which leads to economic losses of animal food production worldwide. In addition, mycotoxins decreased porcine growth performance, immune function, and reproductive performance [2]. Moreover, this well-known xenobiotic is capable to severely damage intestinal health by disrupting intestinal barrier, increasing intestinal permeability, inducing inflammatory responses to human intestinal epithelial cells (IEC_S_) and intensifying the systemic inflammation [3]. Actually, mycotoxins rarely reach the distal small intestine and the colon, thus the proximal parts of the small intestine is the most prominent site of the absorption of mycotoxins and also the major site of antiseptics to act on [4]. However, the distal intestine will also be injured by the basolateral parts of enterocytes after the portal and systemic circulation [5].

It is worth to pay attention that, enterocytes exposed to mycotoxins in gut lumen are particularly susceptible to the contamination of mycotoxins. Mechanistically, the mycotoxin-induced disruption of the gut barrier is an orchestrated process that occurs in association with increased phosphorylated MAPKs and subsequently decreased the expression of tight junction proteins such as Claudins [6], which leads to the damage of barrier integrity [7].

Generally, several mycotoxin-detoxifying agents can be classified into physical removal methods, sequestering (absorbing) agents, and bio-transforming agents that are based on their mode of action. In our research, DMF is the effective additive belonging to biotransformation agents, it is not only able to bind mycotoxins and reduce their gastrointestinal absorption and bioavailability, but also restore a harmonious intestinal micro-environment due to its antioxidant and anti-inflammatory property [8]. The traditional physical methods, such as UR, can decrease the contents of mycotoxins, weaken their toxicity, and in turn improve the integrity of intestinal barrier and permeability [8].

The IECs can also maintain immune homeostasis via contacting with commensal bacteria. If the intestinal epithelial barrier is disrupted, the gut microbiota will pose a risk of infection and inflammatory responses [9]. Considering the important statue of crosstalk between gut microbiota and barrier in gut micro-ecology, it is essential to understand the anti-mildew effect on bacterial communities to realize the full benefits and consequences of dietary anti-mildew technologies.

Several studies have been carried out to investigate the influence of traditional methods on physically removing, in which the innovative disinfectants, such as DMF, were not developed. Moreover, the alteration of gut barrier and microbiota was ignored during the detoxication process. Nowadays, with the development and application of high throughput 16S rRNA sequence analysis, more comprehensive variations of intestinal bacteria caused by the mycotoxin and its disinfectants, such as DMF, could be discovered. In the present study, we aimed at revealing the potential mechanism of DMF that weakened the damage caused by mycotoxins and improved the gut function via modulating intestinal microbiota and barrier. Furthermore, whether the DMF treatment or the TC had a better effect was also compared, in order to make a rational choice between traditional anti-mildew methods and novel anti-mildew additives when detoxicating mycotoxins.

## RESULTS

### Evaluation of DMF and traditional anti-mildew methods in corn

**Figure 1:**
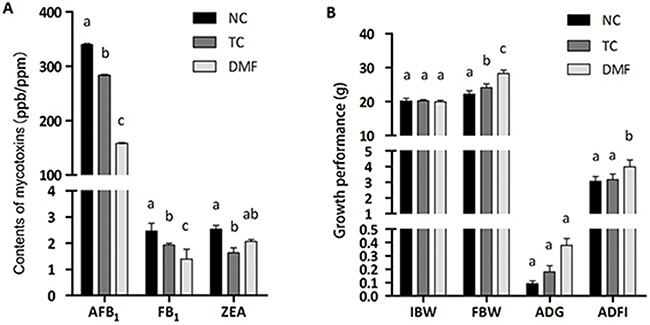
Evaluation of DMF and the traditional anti-mildew method as well as the effect of DMF on growth performance of BALB/c mice The contents of mycotoxins were demonstrated in vertical axis. Different superscript markers between columns indicated significant differences **(A)**. The growth performance was measured with indicators of FBW, ADG and ADFI **(B)**. Values are means ± SE, n = 6. Different superscript lowercase letters within each group means significantly different (*P* < 0.05). AFB1: aflatoxin B1 (ppb); FB1: fumonisin B1 (ppm); ZEA: zearalenone (ppb); TC: traditional control; DMF: dimethyl fumarate; NC: negative control; IBW: initial body weight; FBW: final body weight; ADG: average daily gain; ADFI: average daily feed intake. ADG = (Body weight at the end of the test – Body weight at the start of the test) / (The days of the test period); ADFI = (Feed consumption during the trial period in each duplicate) / (The days of the test period × The number of mouse in each duplicate).

The contents of three kinds of main mycotoxins, which were aflatoxin B_1_ (AFB_1_), fumonisin B_1_ (FB_1_) and zearalenone (ZEA), were all decreased by treating with DMF. The most significantly decreased mycotoxin was AFB_1_, differences among each group were markedly observed (*P <* 0.05). As to FB_1_, DMF still showed the best detoxification effect (*P <* 0.05). However, a significant decrease of ZEA only existed in the traditional control (TC) group when compared to the negative control (NC) group (*P <* 0.05), while the DMF treatment had unmarked effect (*P* > 0.05) (Figure 1A).

### DMF improved the growth performance

DMF significantly improved the final body weight (FBW) (*P <* 0.05) with a non-different initial body weight (IBW) (*P* > 0.05). Meanwhile, the increased level of the DMF group was larger than the TC group when compared with the NC one (Figure 1B). However, there was no variance of the average daily weight gain (ADG) between the DMF treatment and the TC group. In addition, differences in the average daily feed intake (ADFI) between the TC and NC group were also non-significant. Interestingly, significant differences in ADFI (*P* < 0.05) were observed between the DMF and NC group, even between the DMF and TC treatment (Figure 1B).

### DMF improved the intestinal mucosal morphology

**Figure 2:**
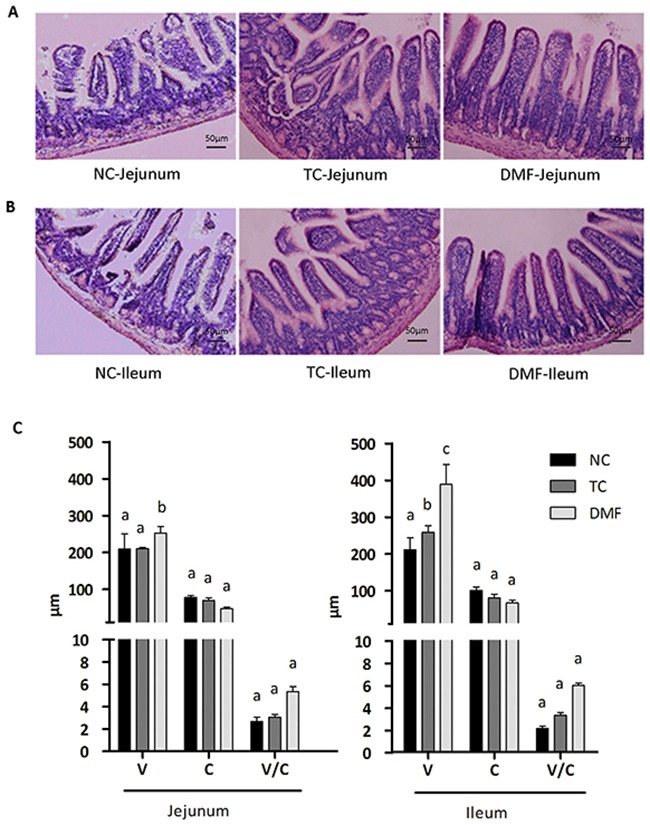
The effect of DMF on the intestinal mucosal morphology To reveal the reason of improved growth performance, the jejunum **(A)** and ileum **(B)** mucosal morphology was observed. The villi lengths (V), the crypt depth (C) and the ratio of the villus length and crypt depth (V/C) were determined in jejunum and ileum **(C)**. Values are means ± SE, n = 6. Different superscript lowercase letters within each group mean significantly different (*P* < 0.05). TC: traditional control; DMF: dimethyl fumarate; NC: negative control.

To reveal the reason for the improved growth performance, the jejunum (Figure 2A) and ileum (Figure 2B) mucosal morphology was observed and determined. The villi length (V) in the DMF group of both jejunum and ileum was significantly higher than the NC group (*P* < 0.05). Although the increased level of the TC group was lower than the DMF one, its increasing was also significant (*P* < 0.05) on the basis of the NC group. In addition, DMF had non-marked influence on the crypt depth (C) and the ratio of the villi length and crypt depth (V/C) both in jejunum and ileum (*P* > 0.05) (Figure 2C).

### DMF increased the expression of Claudin-1 in jejunum and ileum

**Figure 3:**
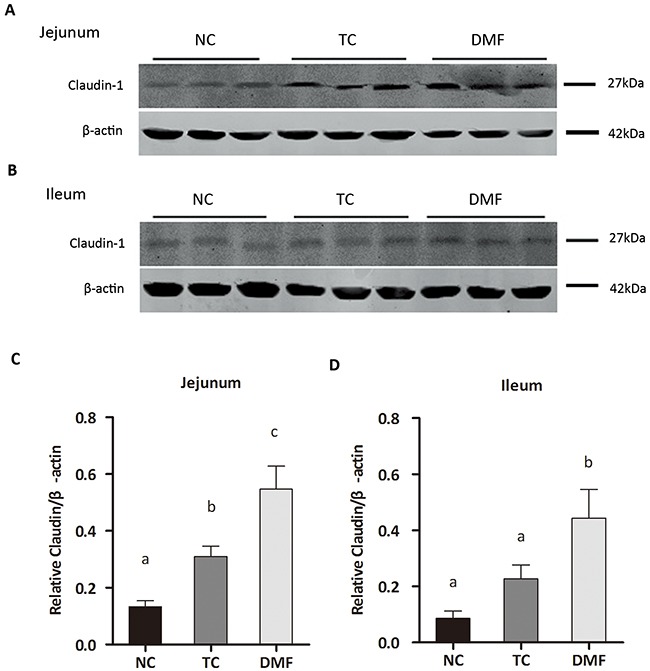
Effects of dietary anti-mildew technologies on the expression of Claudin-1 in jejunum and ileum The abundance of the Claudin-1 protein in mucosal tissues of jejunum **(A)** and ileum **(B)** were determined by Western blot. Representative photos from two independent experiments were shown **(C-D)**. Values are means ± SE, n = 3. Different superscript lowercase letters within each group means significantly different (*P* < 0.05). TC: traditional control; DMF: dimethyl fumarate; NC: negative control.

To evaluate the effect of the DMF treatment to the signal pathway of gut barrier, the expression of mucosal barrier protein, such as Occludin, Claudin-1 and zonula occludens protein-1 (ZO-1) were tested by western blot (Figure 3A–3B). The expression of Claudin-1 in both jejunal and ileal mucosa was significantly increased in the DMF group (*P* < 0.05), when compared with the NC group (Figure 3C–3D). While in the TC group, a marked increase was only detected in jejunum (*P <* 0.05) (Figure 3C). The abundance of Occludin and ZO-1, had no difference in each group of both jejunal and ileal mucosa (*P* > 0.05, data not shown).

### DMF altered the intestinal bacterial richness and diversity in digesta

To further study the mechanism of dietary anti-mildew technologies on improved intestinal mucosal morphology and permeability, the intestinal bacterial richness and diversity in digesta was determined. In total, after size filtering, quality controlling and chimera removalling, 278,713 valid sequences were obtained, with an average of 96,567 sequences, 51,769 sequences and 130,377 sequences separately in per jejunal, ileal and colonic sample. The overall operational taxonomic unit (OTU) number classified at a distance level of 0.03 (97% similarity) was 1,570, among them, 481 was detected in jejunal sample and 402 and 687 were detected separately in ileal and colonic samples, thus, conlonic bacteria community had much more OUT numbers than jejunal and ileal ones. All samples reached a stable plateau based on rarefaction curve analysis, indicating the sampling was sufficient for the majority of the bacterial communities.

The bacterial richness estimated by the Chao-1 estimate (Table 1) showed that, after treating with DMF, higher Chao estimate was detected in jejunum and ileum when compared with the NC group. However, the opposite trend was observed in the colon. In the TC treatment, the bacteria richness in ileum was similar to the control group, but a significant decreasing was detected both in the jejunum and colon.

**Table 1:** Effects of DMF on bacterial communities’ diversity in the jejunal, ileal and colonic digesta ^1^,^2^

Sample ID	Valid sequences	Similarity score ≥ 0.97 ^1^		
		OTU	Chao-1	Shannon
Jejunum_NC	22118	152	119.4	3.95
Jejunum_TC	21021	132	115.6	2.81
Jejunum_DMF	43637	240	196.6	5.70
Ileum_NC	55108	111	55.6	2.09
Ileum_TC	3234	70	117.2	2.44
Ileum_DMF	63660	231	169.1	4.56
Colon_NC	19341	218	191.7	4.76
Colon_TC	27514	200	145.8	3.16
Colon_DMF	23080	216	172.1	5.23

^1^Sequences with similarity scores greater than or equal to 0.97 were clustered into an OTU.

^2^NC: negative control; TC: traditional control; DMF: dimethyl fumarate; OTU: operational taxonomic unit.

The Shannon index analysis (Table 1) indicated that in the TC treatment, the Shannon index was always the lowest no matter compared to the DMF or the NC one. In addition, in digesta of jejunum and ileum, the Shannon index was greater in the DMF group than the NC one, thus the DMF treatment had a statistically significant effect on microbial diversity (*P* < 0.05). In addition, in the large gut, such as the colon, the Shannon index after DMF treatment was similar to both control groups.

### Jejunal bacteria community structure

**Figure 4:**
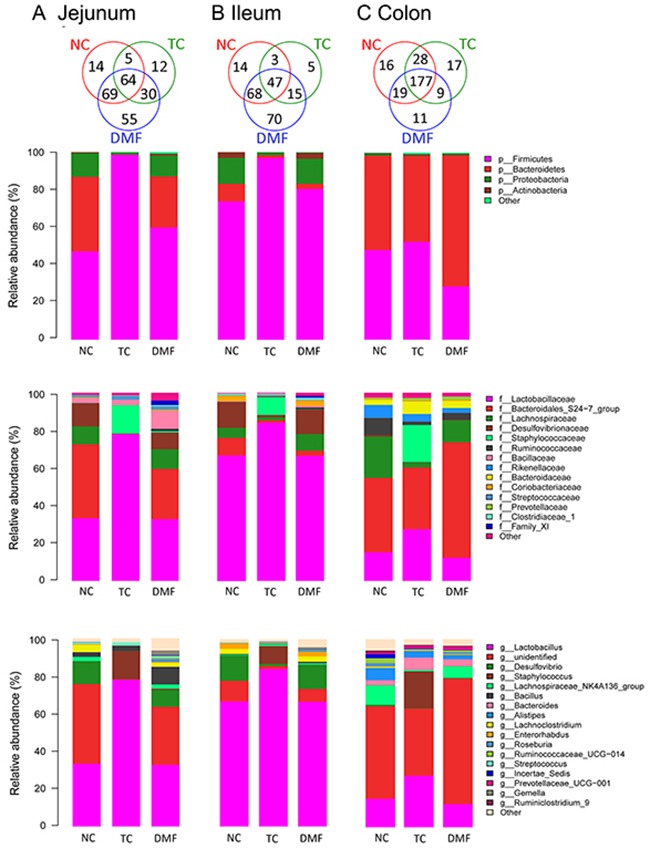
Effects of DMF on jejunum, ileal and colonic bacterial community of BALB/c mice Relative read abundance of different bacterial phylum, families and genus within different communities were detected. The distribution of luminal bacteria in the jejunal **(A)**, ileal **(B)** and colonic **(C)** digesta in different treatments. NC: negative control; TC: traditional control; DMF: dimethyl fumarate.

**Figure 5:**
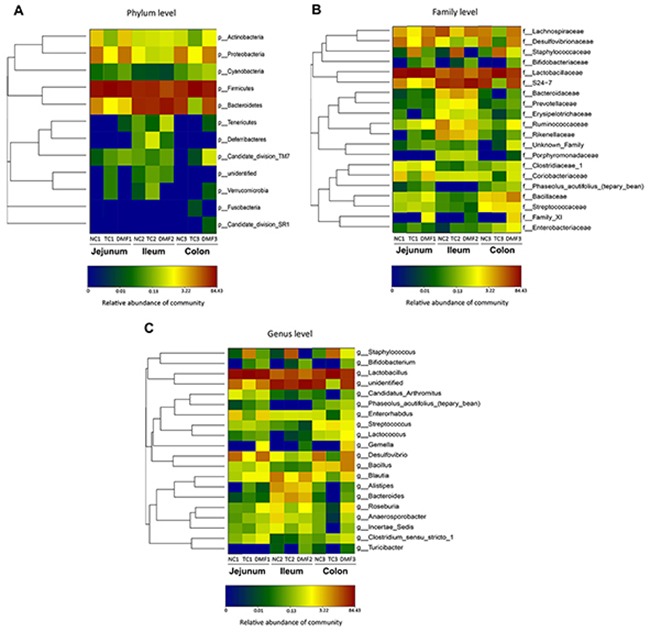
Double hierarchical dendrogram to illustrate effects of DMF on jejunal, ileal and colonic bacterial community of BALB/c mice The distribution of luminal bacteria in the jejunal, ileal and colonic digesta in different treatments were analyzed from phylum **(A)**, family **(B)** to genus **(C)** level, respectively. The bacterial distribution among the samples bacterial phylogenetic tree was calculated by using the neighbor-joining method and the relationship among samples was determined by Bray distance and the complete clustering method. The heatmap plot depicts the relative percentage of each bacteria are depicted by color intensity with the legend indicated at the bottom of the figure. Clusters based on the distance of the eight samples along the X-axis and the bacterial families along the Y-axis are indicated in the upper and left of the figure, respectively. NC: negative control; TC: traditional control; DMF: dimethyl fumarate.

Jejunal bacterial community of three groups shared with 249 OTUs as shown in Venn diagrams, which accounted for 33.29% of the proportion (Figure 4A). Firmicutes, Bacteroidetes and Proteobacteria are dominant phyla in BALB/c mice jejunum, up to 99% of the total jejunal bacterial community (Figures 4A and 5). However, the proportion respectively in each anti-mildew treatment was varied from each other. In jejunum, the alteration trend in both treatment were coincide with each other, but the changes in TC group were more severe. When treated with TC and DMF, abundance of Firmicutes significantly increased from 47.10% to 98.50% and 59.87%, respectively, while abundance of Bacteroidetes decreased from 39.77% to 0.18% and 27.17%. Proportion of Proteobacteria also tended to decrease in TC group from 12.62% to 1.06%, however, no significant decreasing was observed between proportions of DMF and NC group.

At family level, Lactobacillaceae, Lachnospiraceae, Bacillaceae, Streptococcaceae, Ruminococcaceae and Family_XI constituted the main components of Firmicutes, while Bacteroidetes were mainly composed of Bacteroidales_S24-7_group (S24-7) and Proteobacteria were consisted of Desulfovibrionaceae. Lactobacillaceae (33.37%) and S24-7 (39.25%) were the most dominating families in the NC group with different changing tendencies between TC and DMF groups. Lactobacillaceae was significantly increased in TC group from 33.37% to 78.03%, while the proportion of S24-7 dropped dramatically from 39.25% to 0.14% and 26.69% after applying TC and DMF separately. Notably, the proportion of Desulfovibrionaceae and Lachnospiraceae were dramatically decreased in TC treatment, from 12.22% to 0.04%, and from 9.49% to 0.15%, separately. Meanwhile, the increased abundance of Bacillaceae was also significant in the DMF group, which was detected from 2,49% to 9.45%.

Down to genus levels, *Lactobacillus, Desulfovibrio* and *Bacillus* were predominant genera of Lactobacillaceae, Desulfovibrionaceae and Bacillaceae, respectively. Meanwhile, *Lachnoclostridium* and *Lachnospiraceae_NK4A136_group* were both main genera of Lachnospiraceae. Specifically, *Lactobacillus* was dramatically increased in the TC group, which rose from 33.37% to 78.03%, while there was no obvious changing in the DMF treatment. On the contrary, *Desulfovibrio, Lachnoclostridium* and *Lachnospiraceae_NK4A136_group* were all significantly decreased in the TC group with the proportion from 12.18% to 0.04%, from 3.78% to 0.02%, and from 2.25% to 0.05%, respectively, and the degree of this decline was all definitely higher than the DMF treatment. As to *Bacillus*, its significant increase in the DMF group, was up to 9.36% on the basis of 2.45% in the control one. The proportional variation in these bacteria with anti-mildew treatments mainly led to the change of proportion at family level. In addition, about 42.35% jejunal bacteria community were unidentified at genus level.

### Ileal bacteria community structure

About 29.68% OUTs were shared among three treatments of bacterial community in ileum as shown in Venn diagram (Figure 4B). The DMF treatment owned the most unique OTUs than the NC and TC groups. Besides three major bacteria phyla which were Firmicutes, Bacteroidetes and Proteobacteria in jejunum, Actinobacteria was also a main bacteria phyla in ileal contents in NC and DMF groups. They accounted for more than 99% of the total ileal bacteria community.

As to family level, Firmicutes in ileum were mainly composed of Lactobacillaceae and Lachnospiraceae. The TC group had a significant larger proportion of Lactobacillaceae but the lowest proportion of Lachnospiraceae than other two groups. The abundance of Ruminococcaceae, Clostridiaceae_1 and Family_XI, which were all belonged to Firmicutes, was increased and only observed in DMF. The Desulfovibrionaceae belonged to Proteobacteria and the Coriobacteriaceae belonged to Actinobacteria were both dramatically decreased in the TC group compared to the NC one, which from 13.70% to 1.18%, and from 3.03% to 0.09%, respectively. In addition, Bacteroidetes mainly consisted of S24-7, were significantly decreased both in the TC and DMF treatments.

Down to genus level, the *Lactobacillus, Lachnoclostridium and Desulfovibrio* were dominant genera of Lactobacillaceae, Lachnospiraceae and Desulfovibrionaceae, respectively. The proportion of their changes was consistent with the variation of their affiliated family. In addition, a significant increasing from 0.01% to 9.49% in the TC group was detected although there was no marked changes at family level. However, approximate 1.48% to 10.93% of ileal bacteria community was unidentified at genus level.

### Colonic bacteria community structure

The colonic bacterial community was further analyzed to reveal the role of DMF on the large gut microbiota. All sequences were classified and identified from phylum to genus (Figure 4C).

16S rRNA profiles of each experimental group in the colon were very dissimilar even in phylum level distributions. The most abundant phyla associated with the colonic digesta were Firmicutes and Bacteroidetes. It has been detected that in the TC group, the Firmicutes was increased from 48.07% to 52.41%, while after treating with DMF, this phyla dropped to 28.57%. The changes in Bacteroidetes were not significant. However, we could still detect the decreased trend in the TC (from 50.27% to 45.84%) and the increased trend in the DMF group (from 50.27% to 69.80%).

Within these two dominating phyla, there were some significant changes of each treatment at family level when compared to the NC group. The TC treatment decreased the Lachnospiraceae and Ruminococcaceae, both of which belonged to Firmicutes, and the decreased level was from 21.93% to 2.71% and from 9.06% to 1.82% separately. However, Lactobacillaceae was also a major family of Firmicutes, which significantly increased in the TC group, while the decline in DMF treatment was not obviously different from the NC group. S24−7, Bacteroidaceae and Prevotellaceae were all main families of Bacteroidetes, and were increased significantly after treating with DMF. However, the Rikenellaceae were decreased in both anti-mildew treatments.

In the analysis of the bacterial composition down to the genus level, *Lachnospiraceae_NK4A136_group, Incertae_Sedis* and *Roseburia* were dominant genera of Lachnospiraceae, and their respective changes were consistent with the overall change of Lachnospiraceae, that were decreased in both anti-mildew treatments. *Ruminiclostridium_9* and *Ruminococcaceae_UCG-014* had the similar tendency of variation to the family of Ruminococcaceae. In addition, *Lactobacillus, Alistipes, Prevotellaceae_UCG-001* and *Bacteroides* were major genus of Lactobacillaceae, Rikenellaceae, Prevotellaceae and Bacteroidaceae, respectively. What's more, the changes of these genus proportion, with exposing to the TC and DMF treatments, were almost as the same as the changing of family.

## DISCUSSION

The present work was undertaken to compare the different germicidal efficiency of DMF, a novel kind of anti-mildew technology in the BALB/c mice model. In contrast to applying UR which was a traditional and widely-used method on physically removal, adding germicidal additives such as DMF was a more efficient anti-mildew treatment. Our data demonstrated that DMF could detoxify mycotoxins. It is the first time to study the alteration of microbiota and find a promotion of harmonious gut micro-organism especially due to the DMF treatment. Moreover, considering the crosstalk between barrier and microbiota, it's rational for us to infer that DMF can improve the gut barrier and microbiota to gain a more efficient influence on mycotoxin than traditional anti-mildew method.

UR was a common pattern in physical removal methods against mycotoxins and was acted as the TC in our current study. It has been applied to the sterilization of agricultural products which was known to be effective for killing pathogenic moulds that contaminated the surface of grain [10]. These reported germicidal functions of TC [10, 11] was paralleled with our research that three major mycotoxins, such as AFB_1_ (ppb), FB_1_(ppm) and ZEA (ppb), were all significantly deceased in the TC group. Our study also showed that the detoxification function of TC in ZEA was even much better than DMF, indicating that there were some individual functions between TC and ZEA. Consistent with our hypothesis, a previous study found that ZEA possessed the C10–C20 double bond which was essential to induce neutrophil suppression. Interestingly, the present TC treatment was prefer to damage or destroy the bond at least in part, thereby inhibiting the ZEA proliferation as well as alleviating toxic activity of ZEA [12]. On the other hand, a very marked difference of FBW was also showed with the non-significant initial IBW in our study, which was due to the mechanism of TC. The mechanism that disintegrated the mycotoxin structure into less toxic or nontoxic fragments [13] and ameliorating an immunosuppressive effect of mycotoxin [10] was sufficient to explain that the TC decreased mycotoxin contents and promoted the growth performance. However, the efficiency of TC was obviously less than adding DMF. In the TC group, there was even non significant increase not only in ADG but also in ADFI. It seemed contradictory to the effective anti-mildew capacity of TC as former reported to some extent but, actually, TC could inhibit mold growth and the toxin production only on the surface of grain. Thus the mycotoxin ubiquitously throughout the kernel and the endosperm were still existing and adversely affecting the health [10]. In addition, it is necessary for us to consider that germicidal-strength TC treatment has possible detrimental effect on feed quality, if it goes beyond the appropriate method (intensity: 96 W/m2, processing time: 15 h) [10]. A lower improvement may be due to the damage causing to the nutrient quality of feed.

The villus height and the crypt depth are important indicators to reflect the digestive and absorptive functions of the small intestine. Villus is a main micro-structure of nutrient absorption and transportation, and the area of villi will be increased when its height rising that consequently means an improved ability of nutrient absorption [14]. The pathway of transportation in epithelial cells is crypt; its depth mainly reflects the rate of generation of epithelial cells. In addition, the increased rate of mature cells and the enhanced secretary function are due to the decrease of crypt depth [14]. In our research, a significantly increased height of both jejunum and ileum villus was detected in the DMF group when compared to the NC and TC treatments. This marked change in villi demonstrated that DMF could broaden the absorption area of the small intestine and improve the capability of digestion and absorption, more importantly, the effect of DMF was better than the TC treatment. However, even if adding DMF, V/C was no significantly different (*P >* 0.05) when compared with the control groups, which might result from the lower renewal and maturing rate of enterocyte. Accumulated studies reported that the mycotoxin could damage the intestinal structure and cause the toxic effect on gut morphology. Moreover, the mycotoxin also disrupted the tight junction, impaired the intestinal carrier functions, and leaded to an imbalance in the antioxidant system [15]. Paralleled to our observation, there was none inflammatory morphology of intestinal segments with anti-mildew treatments. In addition, the expression of Claudin-1 in the DMF group was significantly higher than that both in NC and TC groups. As an important tight junction protein, Claudin-1 could control epithelial para-cellular pathways [16]. The increased expression of Claudin-1 always indicates a more tightened junction, which downloads the para-cellular permeability [17, 18] and seals the inter-cellular space to prevent the free movement of solutes across epithelial cell sheets [19]. We believe that DMF is a more effective anti-mildew measure due to its unique anti-oxidant and anti-inflammatory ability [8, 20].

Indeed, data from human and animal studies suggested that DMF had anti-oxidant properties. Moreover, the physiological maintenance of gut integrity and the function of intestinal epithelium were positively related to the anti-oxidant activity. Thus DMF might improve the gut barrier with the mechanism of its anti-oxidant property. Recently, it has been demonstrated that DMF reduced the production of reactive oxygen species (ROS) and also reduced the oxidative stress [21]. With ROS constantly touching, the intestinal mucosa was vulnerable to oxidative stress [22]. In addition, DMF leads to the stabilization of NF-E2 related factor 2 (Nrf-2) and activates Nrf-2 dependent transcriptional activity [23]. The function on Nrf-2 not only mediates the neuroprotection and immunomodulation [21], but also promotes the synthesis of detoxifying proteins that are beneficial to gut integrity [23].

Previous study observed that the epithelial disruption was less to be statistically significant in mice at the histological level after treating with DMF [24]. The remission of this inflammatory performance may due to the reduced level of inflammatory cytokines such as tumor necrosis factor-α (TNF-α) and interleukine-1 beta (IL-1β) caused by DMF [24]. TNF-α and IL-1β are the most important cytokines that are present in colon tissues and involved in the pathogenesis of colitis [25]. They promoted the adherence and infiltration of leukocytes to interstitium during acute and chronic colitis, thereby maintaining the chronic inflammation into the caecal and colonic interstitium [26]. Moreover, it was observed that DMF inhibited the nuclear entry of nuclear factor-kappa B 31 (NF-κB31) and degradation of I-kB, both of which were induced by TNF-α [27]. Recent findings demonstrated that NF-κB was not only capable of pro-inflammatory but also of the tissue-protective function [28]. It altered the expression of downstream genes that were responsible for the generation of mediators or proteins in the inflammation associated with experimental colitis, thus were beneficial to gut health [24].

The relation between the gut microbiota and human health is being increasingly recognized. Intestinal microbiota imparts specific function in host nutrient absorption, drug metabolism, maintenance of structural integrity of the gut mucosal barrier, immunomodulation, and the protection against pathogens [29, 30]. Chao-1 estimate indicated the bacterial richness and Shannon index reflected the bacterial diversity. Here, the obtained results showed that the richness of jejunal and ileal bacteria tended to increase after the DMF treatment, suggesting that the DMF intervention might promote the growth of intestinal bacteria. The increased Shannon index in the jejunum and ileum after treated with DMF also showed the more diverse microbiota. These results suggest that DMF has a significantly positive impact on the biodiversity ecosystem of intestine especially when compared with the TC group. Both Chao-1 estimate and Shannon index in TC group were observed to be significantly decreased, thus the intestinal microbiota tended to be single which indicated a negative effect on the healthy gut micro-ecology. The results of the analysis of species composition also confirmed the conclusion above. We found the rising degree of Firmicutes and the reducing degree of Bacteroidetes were all striking in the TC group, while the changes in the DMF treatment were much more moderate. Thus, we suggest that a more reasonable microbial balance with higher diversity is formed. However, the decreased abundance and diversity in ileal bacteria after adding DMF still confused us. We predicted that DMF have certain antimicrobial activities in ileum. Therefore, treating with DMF was indicated to alter gut microbiota, it has been reported that an increase in microbial diversity was related to the enhanced ecosystem stability and the resistance to pathogen invasion [31]. In addition, the identification of toll-like receptor molecular (TLR) in epithelial cells could also be induced, suggesting the stimulated innate immune response and activated PP to secrete antibacterial peptides, which could remove excessive pathogens [32] and was considered to be positive to host health. Therefore, the increased diversity in the intestinal microbiota by DMF is most likely contributed to the improvement of growth performance and the mucosal immune system in intestine [33].

What is crucial, the anti-fungal treatments have been found to improve the intestinal bacterial communities, which might help to reveal the mechanism that improved the growth performance and intestinal barrier function of anti-mildew treatments. Results of our studies with bacteria producing short-chain fatty acids (SCFAs) in the small intestine indicated that bacteria belonging to the *Gemella, Roseburia* and *Bacillus* were stimulated to increase in jejunum and ileum after treated with DMF. Previous studies have shown that *Gemella* was obligatory fermentative, producing either a mixture of acetic and lactic acids or an equimolar molar mixture of acetic acid and CO_2_ depending on the abundance of oxygen [34]. Generally, a slightly acidic intestinal environment can inhibit the colonization and proliferation of pathogens, thus benefiting for gut health. In addition, *Roseburia* prefer to express enzymes favoring the production of butyrate over propionate [35], which was critical in maintaining gut health, especially in growing animals, which have a high butyrate requirement in order to supply the cells of the enlarging intestine. Considering that butyrate not only serves as a source of energy for colonic epithelial cells [36] but is also important to gut physiology, we propose that the rising abundance of *Roseburia* in DMF treatment is beneficial to gut health. *Bacillus* are capable of expressing spore-forming proteins, and recent studies indicated that bacteria genera which were capable of both butyrate production and spore formation might have characteristics expected in probiotic strains. Moreover, the ileal *Bacillus* increment raised by the DMF treatment could be attributed to the increment in ileal barrier function. After supplemented *Bacillus subtilis PB6*, an increased villus height and the abundance of zonula occludin-1 (ZO-1) and Claudin-1 in the ileum were found, indicating a better intestinal development [37, 38], which is consistent with our research.

In the traditional anti-mildew treatment, a drop of *Desulfovibrio* was also obvious in jejunum and ileum. *Desulfovibrio* belongs to a kind of sulfate-reducing bacteria (SRB), which can induce production of lipopolysaccharides (LPS) and are associated with the microbiota inflammatory properties [39]. The decreased level of *Desulfovibrionaceae* in our results meaned a restrained inflammation, which was paralleled to our intestinal morphology trial, the villi height in ileum was improved by DMF and TC group. Thus the anti-mildew treatment could be effective in reducing the inflammatory response by changing the intestinal structure as a result of controlling the *Desulfovibrio* in the upper gut.

Besides the small intestine, the significant increasing of the abundance of *Lactobacillus* was also observed in the colon in TC group. *Lactobacillus* was capable to rapidly establish a complex bacterial community, which prevent host from infected of pathogenic bacteria [40]. This probiotic substance suppressed the expression of proinflammatory cytokine Interleukin 6 (IL-6) and Interleukin 17 (IL-17) and promoted the expression of major tight junction proteins such as Claudin-1 and Occludin [36], which might be correlated with an increment in growth performance in our research. In addition, the abundance of *Bacteroides* was also observed, and this change was limited in the colon, which indicated a specific function of anti-mildew treatments on the lower gut. *Bacteroides* were known to metabolize oligosaccharides of host origin derived from mucins [41], which were predicted to transport oligosaccharides across the outer membrane. *Bacteroides* were also reported to produce both propionate and succinate as terminal products of its metabolism [42]. Furthermore, propionate could not only create an acidic environment but also decreased the expression of virulence factors of pathogens [43].

In addition, a combination of multiple dietary anti-mildew technologies was suggested to reduce toxins in the feed due to their respective effects on the growth performance, intestinal barrier functions and the gut ecology. Based on the theory that UR (TC) could remove the surface mold while DMF could alleviate harmful effects of mold in gut, it is a highlight that a combination of the traditional physical removal method and the innovative additives provide insight into new areas and may lead to new strategies to control mycotoxins in feed. Preferentially, we characterize that the crosstalk between gut microbiota and barrier may be the potential mechanism of DMF to enhance the gut health and food safety. The study we conducted is aimed at a stable gut micro-ecology and is expected to be beneficial to the health improvements in humans.

In summary, we found the DMF was effective in reducing the negative effect of mycotoxins on diets for BALB/c mice. Specifically, we tentatively put forward that fungicides particularly DMF can decrease the oxidative stress and inflammatory responses by modulating the intestinal ecology through regulating the absorption of nutrition, shitting the intestinal microbiota and enhancing the expression of tight junction proteins to improve the intestinal barrier function. What's more, we found a promotion of several prebiotics such as *Lactobacillus, Clostridium, Gemella and Blautia*. They not only inhibit pathogenic bacteria but also derive some beneficial metabolites to maintain and restore the harmony of gut micro-ecology. Thus we rationally rank DMF as a better anti-mildew method.

## MATERIALS AND METHODS

### Animals and treatments

36 BALB/c mice (Beijing Huafukang Biological Company) were divided to three groups, male and female half, each group of three repeats, each repeat contained four mice, 28 d for a trial period. All the mice were housed in cages with a squirrel cage nipple drinkers and a self-feeding trough (room temperature was maintained at 22 ± 2C°, humidity was controlled at 50%-70%). Observations and manure treatments were routinely done. The three groups were individually treated by basal diet (the NC group) (Beijing Huafukang Biological Company), ultraviolet radiation treatment of feed (the TC group) (treated at the animal test base of the National Feed Engineering Technology) and the feed of adding fumaric acid dimethyl ester (0.05%) (the DMF group). Dietary composition and nutrient contents of the basal diet was showed in Table 2. The feed consumption was calculated per cage. The average daily gain (ADG), the average daily feed intake (ADFI) and the final body weight (FBW) were also calculated as indicators. All animals used in this experiment were maintained according to the guidelines of the China Agricultural University Animal Care and Use Ethics Committee. All the experiments were completed at Animal Test Base of the National Feed Engineering Technology Research Center.

**Table 2:** The composition and nutrient levels of basal diets

Items	Content (%)	Nutrient content (%)	
Materials		Moisture	≤8.00
Bran	30.0	CP	≥18.00
Fishmeal	2.00	EE	≥ 4.00
Bean pulp	21.00	CF	≤5.00
Corn	41.00	CA	≤6.50
Soy-bean oil	1.20	Ca	1.20–1.40
Mineral	4.12	P	0.80–1.00
Methionine	0.20		
Choline	0.40		
Vitamin	0.08		
Total	100.00		

### Detection of mycotoxins

The contents of aflatoxin B_1_, Zearalenone, Fumonisins B_1_ in the feed by different mould prevention treatments were quantitatively tested by ELISA kit (Tecna® Elisa Kit, Triesle Italy), with the reference to the national standard. 5g crushed samples (two samples in parallel) were homogenized and 1g of sodium chloride and 25 μL 70% aqueous methanol were added. And then all the samples were oscillated for 3 minutes and centrifuged at 8000 rpm / min for 15 minutes, 100μl Enzyme labeled conjugate was added into the holes of the Elisa plate, each with 50 μL standard sample and 50 μL sample along with 3 times percussion with pipettes, After being mixed, 100μl mixed sample was transferred to the corresponding test hole for 10 minutes reaction and the chromogenic agent was added for 100 μL / hole after 3 times washing, Finally, 50 μL sulfate was added for termination / hole, and the absorbance was measured at 450 nm using an ultraviolet spectrophotometer.

### Tissue sample collection

All mice were sacrificed for later excision of intestinal segments. On d 28, for all mice's jejunum, ileum and colon's intermediate segments, two 3 cm long samples of each segment were quickly removed. After being thoroughly flushed with ice-cold saline to remove the intestinal fluids and chyme, one part was wrapped with aluminum foil and frozen in liquid nitrogen, and then was stored at -80C° for subsequent assay. The other part was preserved in 10% neutral buffered formalin solution for further test and histological analysis.

### Intestinal morphology analysis

All samples were fixed in 4% paraformaldehyde for 48h as previously (Song et al. 2011), samples in jejunum, ileum and colon were fixed and embedded by paraffin. Sections of 2-3μm were cut and stained in hematoxylin and eosin. Each slide was divided into three single segments and the microstructures of jejunum and ileum was analyzed by using an microscope (BX51 type, Olympus Corporation, Japan). The villus height and the crypt depth of 6 randomly chosen villi were measured, and the ratio of villus height and crypt depth of each sample was calculated.

### Protein extraction and immunoblot analysis

The protein levels of tight junction proteins, Occludin, Claudin-1 and ZO-1, were analyzed using immunoblot due to their crucial functions in intestinal mucosal barrier. The jejunal and ileal tissue samples were removed from -80C° and grinded in liquid nitrogen. RIPA / protease inhibitor / phosphatase inhibitor was added to the finely grinding samples to organize small study in an ice bath reaction for subsequent extraction of total protein. The total protein concentration of ileal and jejunal tissue samples was determined by the use of BCA Protein Assay Kit (Pierce Company). 10 μL was added to each sample of the prepared SDS-PAGE for protein electrophoresis. After that same amounts of protein extracts were transferred onto PVDF membranes (Millipore Corporation, USA) and blocked with 5 % skim milk powder solution at room temperature for 2 h. Then the membranes were incubated with diluted primary antibodies of β-actin (1:1,000) and Claudin-1 (1:200) overnight at 4°C. The primary antibodies against Occludin, Claudin-1 and ZO-1 were obtained from Santa Cruz (Santa Cruz, CA), as well as β-actin from Sigma-Aldrich (St Louis, MO). After primary antibody incubation, membranes were carefully washed with 1 × TBST buffer (Tris-buffered saline and Tween 20) for three times, each time 5 min. Then horseradish peroxidase-labeled secondary antibody (1: 10,000) (Beijing Zhongshan Golden Bridge Biotechnology) was added and incubated at room temperature for 1 h, the membranes were washed four times with 1 × TBST buffer, each time 5min, and then washed with PBS solution for 15 min, After incubating with HRP-conjugated secondary antibody, signals were visualized by the LI-COR Infrared Imaging System (Odyssey, Lincoln, NE). Blots were then analyzed by “Quantity One” Software from BioRad Laboratories (BioRad, Hercules, CA), with subsequent calculation of the ratio between the band intensities of β-actin and Claudin-1 in small intestinal segments like jejunum and ileum.

### Composition and diversity of the bacterial communities

Four replicates from per treatment were blended in equimolar ratios based on concentration for PCR amplicons. DNA samples taken from the chyme of jejunum, ileum and colon of BALB/c mice were subjected to 16S rRNA gene sequence-based analysis to examine the characteristic bacterial communities along the mouse intestinal tract, including those presented in the jejunum, ileum and colon. Small fragment libraries whose concentration was more than 30 ng / μL were used for PCR amplification. The samples were detected before treatment after being thawed on ice, centrifuged and mixed thoroughly, Qubit test was used to test the sample concentration. The component and volume of the reagents in PCR amplification system concludes DNA sample X (30ng); Forward Primer (10uM), 2 μL; Reverse Primer (10uM) 2 μL; dNTPs (2.5mM) 4μ L; 10 * Pyrobest Buffer 5μL; Pyrobest DNA Polymerase 3 μL; ddH2O 36.7-X μL (Total 50μL). The PCR procedure condition was setting in 95^°^C for 5 min and then 95^°^C for 30 s and then 56^°^C for 30 s, 25 Cycles and then 72^°^C for 40 s and then 72^°^C for 10 min, finally was ending in 4^°^C. Products were examined on a 2% (150v, 30min) agarose gel. The primers were 338F (5’ACTCCTACGGGAGGCAGCA-3’) and 806R (5’GGACTACHVGGGTWTCTAAT-3’). All procedures were operated according to the manufacture's protocol (Allwegene Company). Two hypervariable regions of 16S rRNA, the V3 and V4 region were used to identify the vast majority of bacteria based on 16S rRNA sequencing. Illumina HiSeq 2000 platform's V3, V4 region through positive and negative reading. Only sequences longer than 50 bp were used for phylotype analysis at the 0.03 OTU level.

### Statistical Analysis

All other data were analyzed using the analysis of variance (ANOVA) procedure of SAS system (version 8.2, SAS Institute, Inc., Cary, NC). The significant difference in the occurrence of growth and other targets among the three groups was tested by Duncan's multiple comparisons test. All indicators were analyzed in each repeat. Statistical results were represented by the mean and standard error, *P* value < 0.05 was considered to be statistically significant.

## Abbreviations

ADFI: average daily feed intake
ADG: average daily weight gain
AFB1: aflatoxin B1
DMF: dimethyl fumarate
FB1: fumonisin B1
FBW: final body weight
IBW: initial body weight
IECS: intestinal epithelial cells
IL-1β: interleukine-1 beta
IL-6: interleukin 6
IL-17: interleukin 17
NC: negative control
NF-κB31: nuclear factor-kappa B 31
Nrf-2: NF-E2 related factor 2
OTU: operational taxonomic unit
TC: traditional control
TLR: toll-like receptor molecular
TNF-α: tumor necrosis factor-α
ROS: reactive oxygen species
S24-7: Bacteroidales_S24-7_group
UR: ultraviolet radiation
V/C: the ratio of the villi length and crypt depth
ZEA: zearalenone.
